# Persistent Viral Reservoirs in Lymphoid Tissues in SIV-Infected Rhesus Macaques of Chinese-Origin on Suppressive Antiretroviral Therapy

**DOI:** 10.3390/v11020105

**Published:** 2019-01-27

**Authors:** Summer Siddiqui, Stefanie Perez, Yong Gao, Lara Doyle-Meyers, Brian T Foley, Qingsheng Li, Binhua Ling

**Affiliations:** 1 Division of Comparative Pathology, Tulane National Primate Research Center, Covington, LA 70433, USA; siqbal1@tulane.edu (S.S.); sperez4@tulane.edu (S.P.); 2 Department of Microbiology & Immunology, Schulich School of Medicine & Dentistry, University of Western Ontario, London, ON N6A 5C1, Canada; ygao387@uwo.ca; 3 Division of Veterinary Medicine, Tulane National Primate Research Center, Covington, LA 70433, USA; ldoyle@tulane.edu; 4 Department of Medicine, Tulane University School of Medicine, New Orleans, LA 70112, USA; 5 Theoretical Biology and Biophysics Group, Los Alamos National Laboratory, Los Alamos, NM 87545, USA; btf@lanl.gov; 6 Nebraska Center for Virology, School of Biological Sciences, University of Nebraska-Lincoln, Lincoln, NE 68583, USA; qli@unl.edu; 7 Tulane Center for Aging, Tulane University School of Medicine, New Orleans, LA 70112, USA; 8 Department of Microbiology & Immunology, Tulane University School of Medicine, New Orleans, LA 70112, USA

**Keywords:** simian immunodeficiency virus, rhesus macaques, lymph nodes, reservoir, antiretroviral therapy

## Abstract

Understanding HIV latent reservoirs in tissues is essential for the development of new strategies targeting these sites for eradication. Here, we assessed the size of latent reservoirs and the source of residual viruses in multiple lymphoid tissues of SIV-infected and fully suppressed rhesus macaques of Chinese-origin (cRMs). Eight cRMs were infected with SIVmac251 and treated with tenofovir and emtricitabine daily for 24 weeks initiated 4 weeks post-infection. Four of the eight animals reached sustained full viral suppression with undetectable viremia. The levels of cell-associated SIV DNA varied in peripheral blood mononuclear cells (PBMCs) and multiple lymphoid tissues, but with higher levels in the mesenteric lymph nodes (MesLNs). The levels of cell-associated SIV RNA also varied in different tissues. The higher frequency of viral RNA detection in the MesLNs was also observed by in situ hybridization. Consistently, the infection unit per million cells (IUPM) in the MesLNs was higher than in PBMCs and other tested lymphoid tissues by quantitative viral outgrowth assay (QVOA). Furthermore, *env* gp120 from tissue SIV RNA was amplified by single genome amplification. Phylogenetic analysis revealed diverse variants from tissues parallel to the viral inoculum in all viral suppressed animals. These results demonstrate that the latency and viral reservoirs in the lymphoid tissues still exist in aviremic macaques under full suppressive therapy. Moreover, the size of viral latent reservoirs differs in various lymphoid tissues with a relatively larger size in the MesLNs.

## 1. Introduction

The viral latent reservoir remains a key barrier in HIV-1 eradication [1]. Although combination antiretroviral therapy (cART) can effectively suppress the viral replication to undetectable levels in peripheral blood, the current antiretroviral drugs are unable to eliminate cellular and tissue latent reservoirs even with the earliest cART during hyperacute HIV infection [2]. Once cART is discontinued, viral productive infections quickly rebound within a few weeks from latent reservoirs in HIV and SIV infection. The exact sources of viral rebound have not been identified [3,4]. Lymph nodes (LNs), the spleen, gut-associated lymphoid tissues, the reproductive system, and the central nervous system are proposed to account for viral persistence [5]. One recent study showed that the B cell follicles of lymph nodes harbor productive viral replication in elite SIV-controlling macaques due to less access by SIV-specific cytotoxic T cells [6]. HIV also persists in follicular dendritic cells by trapping viable virions on their surface and in follicular helper CD4^+^ T cells inside the germinal center of the LNs in HIV-infected patients with suppressive cART [7,8] or in the lymphoid tissues in aviremic patients on cART [9], indicating that the low-level ongoing virus replication may be another source of plasma virus rebound after cessation of ART.

To identify and characterize cellular and tissue latent reservoirs or determine whether there is an ongoing low-level replication in lymphoid tissues on suppressive ART, multiple methods have to be used to corroborate each other, because different assays such as PCR-based, quantitative viral outgrowth assay (QVOA), and in situ hybridization have different advantages and drawbacks. However, the combination of these methods has not often been used in determining whether lymph nodes harbor replication-competent viruses and whether there is ongoing active replication in tissues under suppressive cART despite an undetectable viral load in peripheral blood. Thus far, by either the assays of PCR-based or in situ hybridization, lymphoid tissues, and lymph nodes in particular, have been found to contain high viral DNA loads [10,11].

Used in combination, the nucleoside reverse transcriptase inhibitors (NRTI), tenofovir and emtricitabine, are still the basis of antiretroviral drugs in use with integrase or proteinase inhibitors for treating HIV infection. Tenofovir and emtricitabine have also been used on rhesus macaques of Chinese-origin (cRMs) for investigating latent reservoirs in the CNS [12] and mucosal immune responses, where ~ 60% of animals can reach full viral suppression in peripheral blood when only these two drugs were available for treatment [13]. It is relatively easier and quicker to suppress plasma viral loads (pVL) in SIV infection in cRMs to undetectable levels by cART [14,15], as compared with SIV infection in Indian rhesus macaques. This two-drug combination therapy in cRMs could suppress plasma viral load to undetectable levels for latency studies [13].

In this study, we treated SIVmac251-infected cRMs with tenofovir and emtricitabine to examine the size and the distribution of SIV latent reservoir(s) in lymphoid tissues. Four of the eight animals reached sustained undetectable plasma viral loads on ART. At the end of ART, cell-associated viral DNA was detected in PBMCs and all the lymphoid tissues examined (axillary, inguinal, mesenteric lymph nodes and spleen). Cell-associated viral RNA was also detected in the lymphoid tissues examined as above but not in PBMCs by qRT-PCR. The presence of SIV RNA+ cells in the mesenteric lymph node tissues was confirmed by using in situ hybridization, indicating that there were persistent viral reservoirs in lymphoid tissues despite aviremia. Furthermore, QVOA revealed that different lymphoid tissues contained replication-competent viruses, of which the mesenteric lymph node tissues had the highest number of infection units. In addition, the viral variants isolated in the lymphoid tissues did not show specific compartmentalization by phylogenetic analysis but were similar in diversity to the swarm virus in the inoculum. Our data demonstrate that the mesenteric lymph nodes are likely to be one of the sources of viral reservoirs with possible continuous active viral replication. Although it leads to sustained suppression of peripheral viremia by the two reverse transcriptase inhibitors (RTi), a larger group of animals with earlier and longer treatment over 6 months and a novel antiretroviral regimen can be tested in the SIV/cRM/cART model for further reduction of these reservoirs.

## 2. Materials and Methods

### 2.1. Animals and Virus Inoculation

Eight rhesus macaques of Chinese-origin (*Macaca mulatta*) were studied. The animals were housed at the Tulane National Primate Research Center (TNPRC) and maintained in accordance with the standards of the American Association for Accreditation of Laboratory Animal Care and the “Guide for the Care and Use of Laboratory Animals” prepared by the National Research Council. The protocol of all studies was approved by the Tulane Institutional Animal Care and Use Committee (IACUC, P0121R-2015). The animals were sero-negative for SIV, simian D retrovirus and simian T-cell leukemia virus prior to SIV inoculation. The animals were inoculated intravenously with 100 TCID50 of SIV grown in culture from SIVmac251.

### 2.2. Antiretroviral Therapy

Beginning 4 weeks post-infection, each animal received the reverse transcriptase inhibitors (R)-9-(2-phosphonylmethoxyypropyl) adenine (PMPA, tenofovir; 20 mg/kg) and beta-2′,3′ dideoxy-3′-thia-5-fluorocytindine (FTC, emtricitabine; 40 mg/kg) daily by subcutaneous injection. ART was continued through the end of the study for 24 weeks. The animals were then euthanized and necropsied for extensive tissue collection for the reservoir evaluation. Tenofovir and emtricitabine were generously provided by Gilead Sciences, Inc. (Foster City, CA, USA) via Material Transfer Agreements.

### 2.3. Quantification of Viral RNA in Plasma

The blood samples were collected in ethylenediamine tetraacetic acid (EDTA)-treated tubes and the plasma was separated from whole blood and stored at −80 °C until use. The viral RNA (vRNA) was extracted from the plasma using the high pure viral RNA kit (Roche; Indianapolis, IN, USA). The SIV plasma viral loads (pVLs) were quantified by real-time quantitative PCR (qPCR) assay by the Pathogen Detection and Quantification Core of Tulane National Primate Research Center as described elsewhere [16] using the primers and probes for conserved *gag* region of SIVmac239 and SIVmac251.

### 2.4. Quantification of Cell-Associated SIV DNA and RNA from Blood and Lymphoid Tissues

The RNA from peripheral blood mononuclear cells (PBMCs) and lymphocytes obtained from the lymph nodes and spleen during the necropsies was isolated with TRIzol^®^ Reagent (Thermo fisher scientific, Waltham, MA, USA) according to the manufacturer’s protocol with slight modifications. The sample was separated with chloroform and the aqueous phase was treated with isopropanol to precipitate the RNA; the interphase was used to precipitate the DNA. The TaqMan Gene Expression Master Mix (LifeTechnologies, Inc., Carlsbad, CA, USA) was used in the qRT-PCR reaction. The levels of the cell-associated (CA) SIV DNA and CA SIV RNA viral loads were determined using methods described in detail elsewhere [14].

### 2.5. SIV RNA Detection in Lymphoid Tissues Using in Situ Hybridization

Axillary lymph nodes (AxLNs), mesenteric lymph nodes (MesLNs), and spleen tissues were collected at necropsy, fixed in Z-fix and embedded in paraffin. Six-micrometer-thick sections of tissue were cut and mounted on charged glass slides (Fisher Scientific, Waltham, MA, USA). Isotope ^35^S labeled sense or the antisense riboprobes of SIV were used as described previously [17]. The slides were exposed for 2 weeks.

### 2.6. Isolation and Purification of Resting CD4^+^ T Cells from Blood and Tissues

PBMCs were purified from whole blood via Hypaque–Ficoll gradient centrifugation. The CD4+ T cells from PBMCs as well as lymphocytes isolated from LNs and the spleen were negatively selected to remove CD8^+^ T cells, B cells, monocytes, NK cells, and granulocytes cells using a cocktail of biotin-conjugated antibodies and anti-biotin micro magnetic beads using a non-human primate microbeads CD4^+^ T cell isolation kit, (Milltenyi Biotech, Auburn, CA, USA). The purified CD4^+^ T cells were further separated by non-human primate microbeads, anti-CD25 and anti-HLA-DR antibodies for resting CD4+ T cells. The resulting resting CD4^+^ T-cell population generally reached >95% purity.

### 2.7. Quantitative Viral Outgrowth Assay (QVOA)

Highly purified resting CD4^+^ T cells were activated in the presence of 0.5 μg of PHA/mL to stimulate the virus production from latently infected cells. The purified resting cells were carefully counted and suspended to 1 × 10^6^ cells/mL in PHA containing media. The purified cells were cultured in duplicate using 5-fold limiting dilution, ranging from 1 × 10^6^ to 3.2 × 10^2^ cells/mL, respectively. On day 2, PHA was removed with medium containing IL-2. The cells were co-cultured with 1 × 10^5^ CEMx174 for two weeks. The CEMx174 cells served to expand the virus released from infected cells as previously described [18]. The culture supernatant was collected weekly, and fresh medium was added to the culture. Culture supernatants were stored at −80 °C in 1.5-mL aliquots. The frequency of cells harboring replication-competent viruses was determined by limiting dilution assay statistics and expressed as the infectious units per million (IUPM) that was calculated using the IUPMStats v1.0 infection frequency calculator (available online: http://silicianolab.johnshopkins.edu) [19].

### 2.8. SIV env Sequence Analysis

Total RNA was extracted from PBMCs at different time points, lymphocytes from LNs, and spleen tissues at necropsies. The extracted RNA was reverse transcribed into cDNA using the SuperScript III reverse transcriptase enzyme kit (Life Technologies, Carlsbad, CA, USA) according to the manufacturer’s protocol. cDNA was then used for single genome amplification (SGA). Platinum PCR SuperMix High Fidelity kit (Life Technologies) was used for nested PCR following the manufacturer’s protocol. The initial PCR cycles (1st round) were carried out using the following primers: 1st round (Fwd: 5′-CTA TAA TAG ACA TGG AGA CAC CCT TG-3′; Rev: 5′-CTT CTT GCA CTG TAA TAA ATC CCT TCC-3′) and 2nd round (Fwd: 5′-ATG CAA CCA CTC CAG AAT CGG C-3′; Rev: 5′-GGA ACT CTC CTC TGC AAT TTG TCC-3′). Both rounds were amplified with the same cycling conditions: 94 °C for 2 min then 40 cycles of 94 °C for 30 s, 55 °C for 30 s, and 72 °C for 1 min. The size of the resulting product was ~1500 base pairs. The PCR product was purified using the DNA and PCR clean kit (Qiagen DNA purification kit, Hilden, Germany) according to the manufacturer’s protocol and used for sequencing. The raw sequences were assembled using Sequencher 5.4.1 sequencing software (Gene Codes Corporation, Ann Arbor, MI, USA). All assembled sequences were manually corrected for individual ambiguous nucleotide errors and to eliminate any multiple templates. The SIV *env* sequences were aligned with SIVmac239 and SIVmac251 as reference sequences. The sequences of the KC522 series of SIVmac251 inoculum were also included in the analysis [20]. Molecular evolutionary genetics analysis (MEGA) version 7.0 and the maximum likelihood (ML) method were used for the generation of phylogenetic tree analysis [21].

### 2.9. Nucleotide Sequence Accession Numbers

Viral *env* sequences were deposited in GenBank under accession numbers MH745243-MH745367.

## 3. Results

### 3.1. Dynamics of SIV Plasma Viral Load during SIV Infection and Antiretroviral Therapy

**Figure 1:**
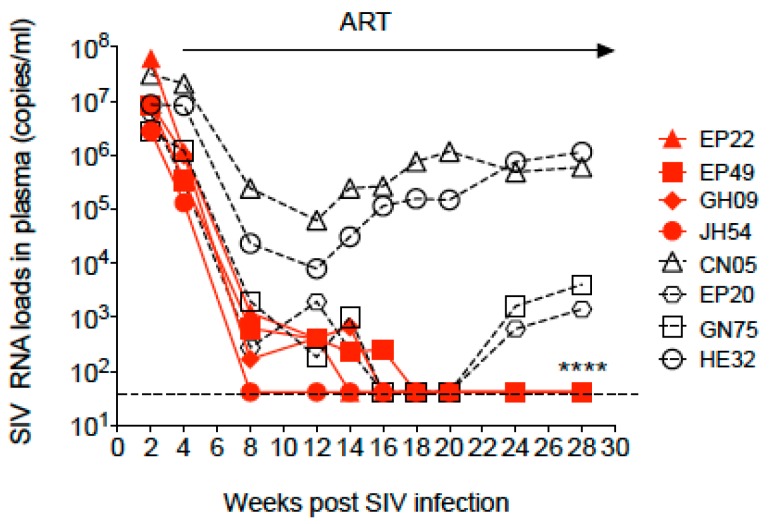
Dynamics of the SIV RNA load in plasma during SIV infection and combination antiretroviral therapy (ART) in Chinese-origin rhesus macaques. ART was initiated at week 4 post-SIV infection and continued for 24 weeks. The arrow indicates the period of continuous ART. The dashed line shows detection of the limitation (41 copies/mL). * time of euthanasia of the four animals with undetectable plasma viral loads.

Eight cRMs were infected with SIVmac251. Viremia peaked at 2 weeks post-infection to the median of 8.5 × 10^6^ copies/mL (ranging from 2.7 × 10^6^–6 × 10^7^ copies/mL) with the highest pVL in animal EP22. By the time of the initiation of ART at week 4 post-infection, the median pVL was reduced to 1 × 10^6^ copies/mL (ranging from 1.3 × 10^5^–2 × 10^7^ copies/mL). ART was administered daily for 24 weeks (Figure 1). During ART, six animals reached undetectable plasma viral loads (pVL) at different time points, while two of them (EP20 and GN75) had detectable pVL again by the end of ART. The remaining four animals, EP22, EP49, GH09 and JH54, maintained undetectable levels until the time of euthanasia. Two animals, CN05 and HE32, had decreased viral loads in the 1st month of ART but maintained levels of 10^4^–10^5^ copies/mL similar to that of untreated animals as previously observed [13], indicating mild or no response to ART.

### 3.2. Cell-Associated SIV DNA in Blood, Peripheral LNs, Mesenteric LNs and Spleen

**Figure 2:**
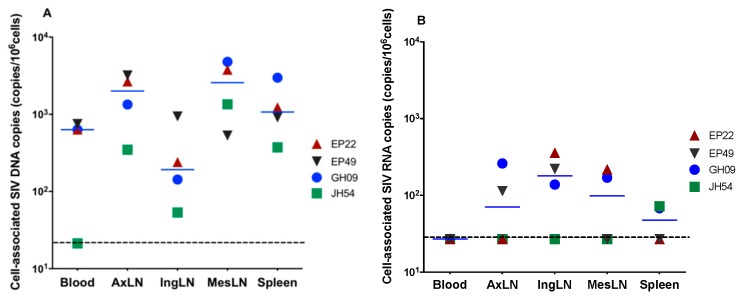
Levels of cell-associated SIV DNA (**A**) and RNA (**B**) in blood, axillary lymph nodes (AxiLNs), inguinal lymph nodes (IngLNs), mesenteric lymph nodes (MesLNs) and the spleen at the end of 6 months of ART in four animals with full viral suppression in peripheral blood. The dotted line indicates the limit of detection.

We then focused on the four animals that reached full viral suppression to assess the size of the viral reservoirs in different compartments. Cell-associated (CA) SIV DNA were detected in all the tissues collected, including PBMCs, the lymphocytes from lymph nodes (axillary, inguinal and mesenteric) and the spleen (Figure 2A). The CA SIV DNA levels ranged within 10^2^–10^4^ copies/10^6^ cells for all the animals with the highest levels (4.8 × 10^3^ copies/10^6^ cells) in GH09 in the MesLNs. Inguinal lymph nodes (IngLNs) had the lowest median CA SIV DNA (1.9 × 10^2^ copies/10^6^ cells) and the highest median CA SIV DNA in the MesLNs (2.6 × 10^3^ copies/10^6^ cells). JH54 had less than 100 copies/10^6^ cells of CA SIV DNA in blood and inguinal lymph nodes.

### 3.3. Cell-Associated SIV RNA in Blood, Peripheral LNs, Mesenteric LNs and the Spleen

To determine whether there is active SIV replication in lymphoid tissues, the CA SIV RNA levels were quantified using qRT-PCR. SIV vRNAs were detectable in variant tissues from all four aviremic animals. EP22 showed the highest SIV RNA levels in IngLNs. EP49 had detectable CA SIV RNA levels in AxLNs and IngLNs. GH09 had detectable CA SIV RNA levels in all the LNs as well as in the spleen, while CA SIV RNAs were only detected in the spleen of JH54. Interestingly, all four animals had no detectable CA SIV RNA levels in PBMCs, indicating the effectiveness of the ART on peripheral blood (Figure 2B).

### 3.4. SIV RNA+ Cells Were Detected in the Lymph Node Tissues of Aviremic Animals by in Situ Hybridization

**Figure 3:**
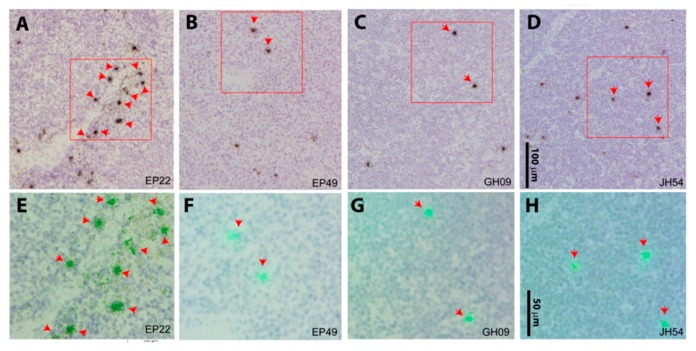
SIV viral RNA positive cells from mesenteric lymph node LN tissues. Viral RNA+ cells were readily detected in mesenteric LN tissues from all four aviremic animals using ^35^S riboprobe in situ hybridization. The SIV RNA-positive cells were overlaid by silver grains (black in transmitted light shown in the upper panel (A to D represent animals EP22, EP49, GH09, and JH54), and green under epipolarized light shown in the lower panel (E to H in correspondence with A to D) after radioautography for 14 days. The red box on the upper panel was highlighted at a higher magnification in the corresponding lower panel. Scale bars: 100 μm for the upper panel, 50 μm for the lower panel.

To validate the qRT-PCR results, isotope ^35^S labeled anti-sense riboprobe in situ hybridization (ISH) was used for vRNA+ cells detection in AxLNs, MesLNs, and spleen tissues. SIV RNA+ cells were readily detected in the MesLNs and spleen tissues in all four aviremic animals on suppressive ART, with higher frequency in the MesLNs than spleen tissues (Figure 3 and Figure S1), while SIV RNA+ cells were rarely detected with a few positive cells in entire tissue sections in AxLNs and in the spleen (Figure S1). The isotope ^35^S labeled sense riboprobe was used as a negative control and we did not detect signals (Figure S2). Additionally, the anti-sense riboprobe hybridized to SIV-uninfected macaque MesLN tissues was used as a negative control and we did not detect any signal either (Figure S2).

### 3.5. The Size of Viral Latent Reservoir Was Higher in Mesenteric LN Than Other Tested Tissues

**Figure 4:**
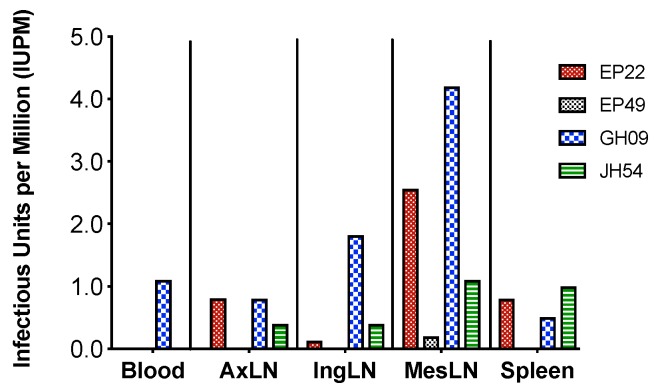
The size of replication-competent viral reservoirs in different tissues at the end of ART in the four animals with full SIV suppression in peripheral blood. The missing bars indicate undetectable levels of IUPM, e.g., IUPMs were undetectable in the blood of EP22, EP49 and JH54.

We further used the viral outgrowth assay to evaluate the size of viral reservoirs of the replication-competent viruses in the collected tissues. When compared with blood, spleen and peripheral LNs, MesLNs had the largest sized reservoirs with the range of 1–4.2 IUPM in resting CD4+ T cells by the end of ART in EP22, GH09 and JH54 except EP49 which had low IUPM. It was noteworthy that MesLNs were the only tissues that showed detectable IUPM in EP49 (Figure 4). Although the levels in each individual had different absolute numbers, this result was concordant with the findings by molecular assays.

### 3.6. Viral Variants in Different Tissues

To assess SIV variants distribution in different compartments including blood, LNs and spleen, we analyzed SIV *env* gp120 sequences using the single genome amplification (SGA) of viral RNA and determined the phylogenetic relationship between the blood and lymphoid tissues. Due to the undetectable plasma viral RNA, virion gp120 RNA sequences were not available in the plasma and PBMCs of all four fully suppressed animals and some lymphoid tissues with an extremely low level of viral RNA at the end of ART when the animals were euthanized. Therefore, we sequenced SIV *env* gp120 in PBMCs at the peak (day 14) and day 30 post-SIV infection and also from the lymphocytes of the AxLNs, IngLNs, MesLNs and spleen collected at necropsies when the PCR results were positive.

**Figure 5:**
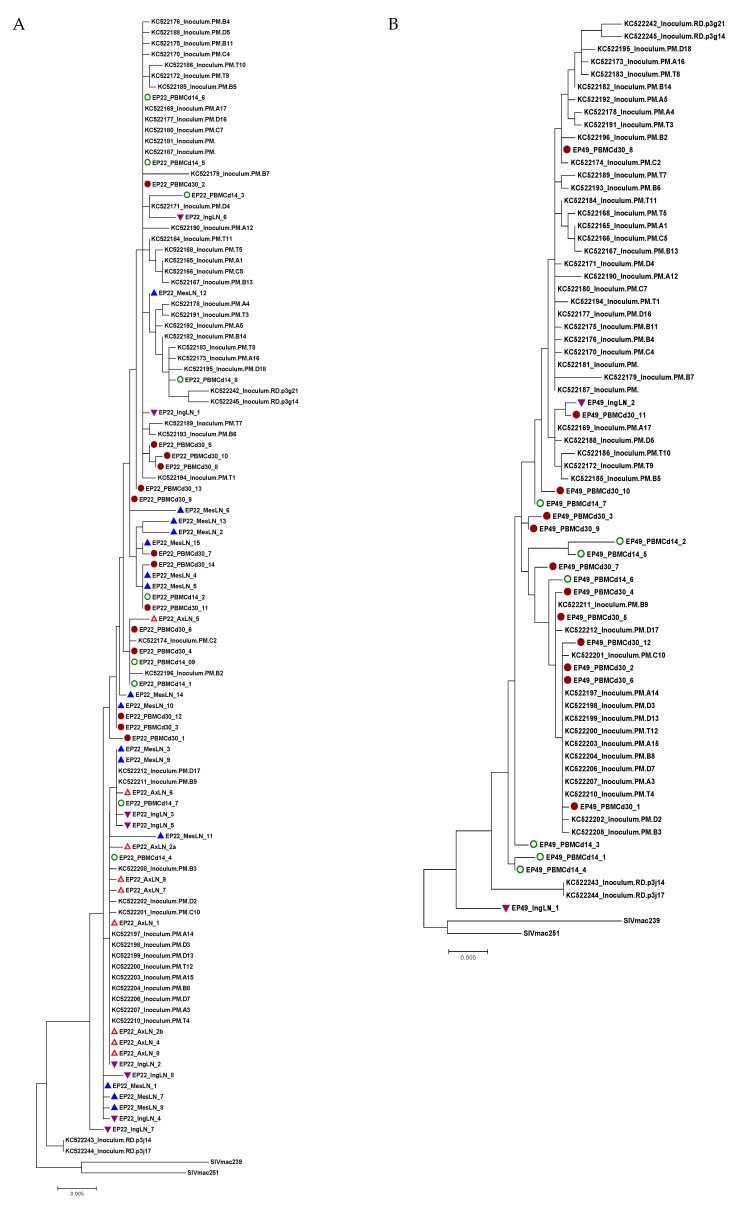

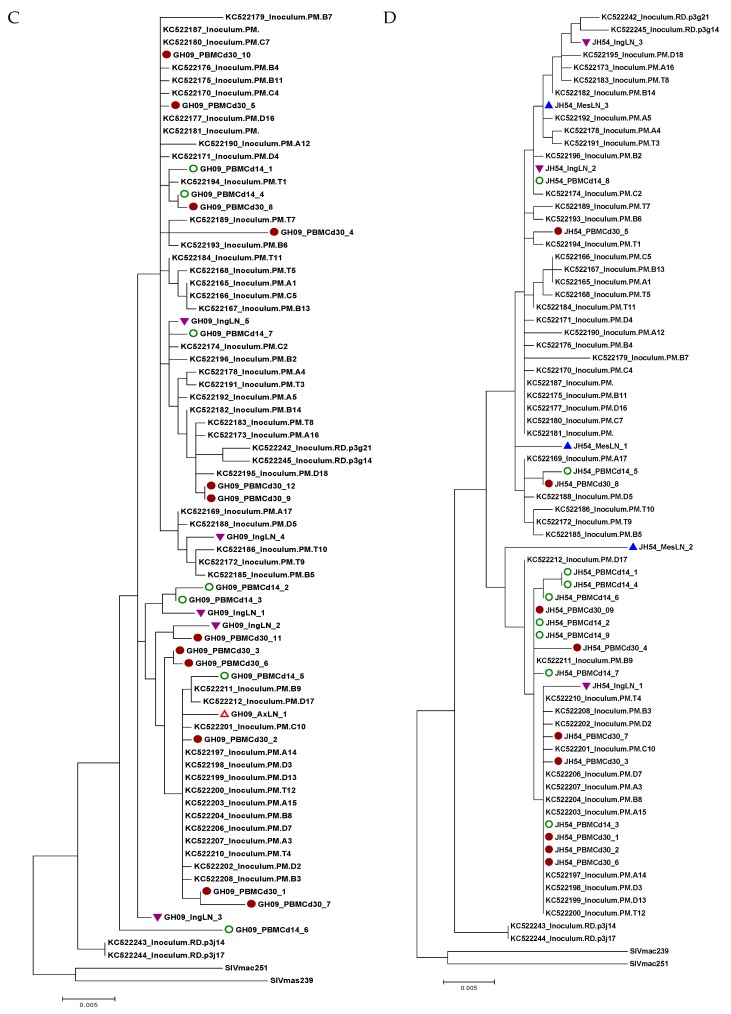
Phylogenetic trees of SIV *env* gp120 sequences obtained from viral RNA from multiple lymphoid tissues from the four animals with an undetectable plasma viral load during therapy. Each tree represents each animal (**A**: EP22, **B**: EP49, **C**: GH09 and **D**: JH54). The tree scale bar indicates the number of substitutions per site. Green circle: PBMC at day 14 post-infection; Solid round circle: PBMC at day 30 post-infection; Red triangle: Axillary lymph node (AxLN); Inverted purple triangle: Inguinal lymph node (IngLN); Blue triangle: Mesenteric lymph node (MesLN). The sequences of the KC522 series of the SIVmac251 stocks [21}, SIVmac239 and SIVmac251, were obtained from GenBank for comparison.

We obtained SIV *env* gp120 sequences from EP22 from all the lymph nodes at necropsy but not from the spleen, whose sequences had no significant compartmentalization clusters from PBMCs at day 14, day 30 p.i. and all tissues during suppressive ART (Figure 5A). For animal EP49, we obtained only two isolates from the IngLNs from 48 reactions of the SGA amplification, and no positives were found in the PBMCs, AxLNs, MesLNs and spleen at the end of ART (Figure 5B). Similarly, we recovered one isolate from the AxLNs and four isolates from the IngLNs in GH09 (Figure 5C). For JH54, we isolated three variants from each tissue of the IngLNs and MesLNs without positives in the AxLNs and spleen (Figure 5D). In each animal, the isolates from the different tissues were not significantly different from those obtained from PBMCs at day 14 and day 30 post-infection before ART. The isolates were also not different from the Tulane SIVmac251 virus stock lineages of KC522 series [20].

## 4. Discussion

The establishment of a non-human primate model of HIV latency is essential for studies of viral tissue reservoirs. Here, with the availability of tenofovir and emtricitabine generously provided by Gilead, we treated eight cRMs and half of them reached full viral suppression with a sustained undetectable plasma viral load. The latency status in four of these cRMs allowed us to study the size, viral diversity, location and distribution of viral latent reservoirs or potential low-level replication in different lymphoid tissues. Although it has been demonstrated that lymph nodes have the most latently infected memory CD4+ T cells among tissue reservoirs in HIV-1-infected individuals and SIV-infected macaques on fully suppressive cART, we found that the viral reservoir distribution in different lymphoid tissues were at different levels in this SIV/cRM/ART model, parallel to other SIV-susceptible NHP models, making it serve as an equally important model for HIV persistence and cure research.

Molecular-based assays have been widely used to evaluate viral persistence in HIV-1 basic research and clinical settings. The total HIV-1 DNA is a valuable marker reflecting HIV persistence as this contains all forms of residual HIV in blood and tissues that contribute to viral production, HIV pathogenesis and immune activation [22,23]. Cell-associated HIV-1 RNA may be able to generate mRNAs and proteins that play a role in pathogenesis even though it may not contain replication-competent viruses. These parameters are also frequently used in the NHP model. In this study, we found that the levels of CA SIV DNA in tissues were measurable, indicating that viral reservoirs exist in all of the tested tissues with the highest in the MesLNs. These results are consistent with findings from other groups, which showed the highest levels of SIVgag RNA in the MesLNs among tested lymphoid tissues in RT-SHIV-infected rhesus macaques on ART [24], and the highest vRNA levels in both superior and inferior MesLNs [10]. Moreover, using in situ hybridization, we showed consistent results that the MesLNs had detectable SIV RNA. Furthermore, by using the gold standard QVOA, we confirmed that that the viruses harbored in the MesLNs are replication-competent. Thus, by using PCR-based assays, in situ hybridization technology and gold standard cell culture QVOA, we found that mesenteric lymph nodes harbored the most replication-competent viruses compared to other peripheral LNs and spleen.

The size or level of the viral reservoir depends on the time of initiation of cART, the length of the treatment period, the combination of different categories of antiretroviral drugs, individual biological responses and other factors. Currently, there are three possible mechanisms of viral persistence and reservoirs under suppressive cART, (1) long-live resting memory CD4+ T cells [25,26,27], possible tissue macrophages [28,29,30,31] and other cell types [7,32]; (2) the clonal expansion of CD4+ T cells [33,34,35,36]; and (3) extremely low levels of ongoing productive replication or active reservoir. The latter possible mechanism is still controversial with both evidence for [9,37,38,39] and against [40,41] it. It is well understood that peripheral blood reservoirs do not reflect the status in different tissues. Conceivably, undetectable viral loads in circulating blood do not mean that there are no viruses anywhere, because the limit of detection is above zero. This is also because antiretroviral drugs cannot penetrate all body sites equally with the existence of sanctuary sites.

Although we did not test tenofovir (TFV) and emtricitabine (FTC) concentrations in the LNs and spleen, it has been shown that the average concentrations of TFV-diphosphate were 80% lower and those of FTC-triphosphate were 66% lower in lymph nodes compared to those of PBMCs [38]. Therefore, productive replication is unexcluded in the LNs, particularly in the MesLNs. Since MesLNs play a pivotal role in the center of immune anatomy [42], the observed high levels of SIV RNA in the MesLNs are likely due to their particular sites and functions, which may deliver microbial translocation from leaking gut with constant activation of SIV target cells.

The analysis of viral compartmentalization and evolution can provide valuable information on whether there is productive viral replication under suppressive cART. Observing viral evolution would clearly indicate active viral replication. However, it is extremely difficult to prove whether there is ongoing evolution and replication under cART. Within HIV-infected humans, the extreme low levels of viral loads due to effective cART therapy usually result in difficulty when it comes to assessing virus evolution. By the time that the treatment is started, there is already considerable diversity in the virus population, thus it is very challenging to analyze any further viral evolution as small populations cause much slower evolution. However, the lack of evidence of viral evolution does not mean that viral replication production is not happening.

In this study, viral isolates were closely clustered with the TNPRC virus stock SIVmac251 such as the KC522171-PM series that were sequenced in a previous study [20]. The inoculum had some diversity as a swarm virus, as do most SIV stocks that are used for vaccines or other studies. There were not any detectable tissue-specific lineages from the phylogenetic analysis. Almost all of the variability was in one small region in the V1V2 loop as commonly seen for the SIVmac viruses, SIVsm and HIV-2, in which the V1–V2 region is far more variable than in other regions. These viruses evolve differently in the *envelope* than HIV-1 and other viruses. However, the small differences in our sequences were not significant, and there had not been much evolution from the inoculum. Our results were consistent with observations by others that no viral evolution was found in the LNs of SIVmac-infected macaques on cART [43]. Again, if the virus is fairly well suppressed by cART, it might be unable to considerably evolve. Significant evolution is expected more in untreated animals; for treated animals and elite controllers, it is only expected over many years of infection [44,45].

Overall, the phylogenetic tree analysis did not demonstrate compartmentalization within the same animal, indicating that the viruses in the LNs isolated at 24 weeks of ART were possibly derived from variants which existed before ART started. It is also likely that 24 weeks of ART with only two RTi were not effective enough to fully suppress the extremely low level of virus replication in tissues. Since we did not have longitudinal samples from the same tissue, we were unable to directly address the viral evolution issue using only this assay. However, in combination with the results from QVOA, the production of residual viruses was likely from the MesLNs.

## 5. Conclusions

Together, we found that SIV-infected cRMs with full viral suppression in blood can be used for SIV latency studies. Lymphoid tissues, particularly MesLNs, are viral tissue reservoirs of latent infection without the exclusion of possibly extremely low ongoing viral production. Treatment periods lasting longer than 6 months are likely needed for full suppression in tissues even if blood viremia is consistently undetectable. Since the local microenvironment of the lymphoid tissues may contribute to the uneven viral distribution, when evaluating viral reservoirs, multiple tissues need to be examined. In addition, given that the sample size in this study is relatively small, we expect that future studies with larger groups of animals receiving a potent triple-drug regimen for longer treatment will reduce the size of the reservoirs in the MesLNs and other lymphoid tissues. Multiple methods, in combination with PCR-based, in situ, and cell culture assays, may be needed to determine the effectiveness of new approaches for SIV and HIV eradication.

## Supplementary Materials

The following are available online at http://www.mdpi.com/1999-4915/11/2/105/s1, Figure S1: SIV viral RNA positive cells in axillary lymph node LN and spleen tissues, Figure S2: Positive and negative control for SIV RNA in-situ hybridization.
