# Supplementary material for: Persistent Viral Reservoirs in Lymphoid Tissues in SIV-Infected Rhesus Macaques of Chinese-Origin on Suppressive Antiretroviral Therapy
Source: Viruses. 2019 Jan 27;11(2):105. doi: 10.3390/v11020105 (PMC6410399; doi:10.3390/v11020105)
Supplement: Supplementary file 1 [file viruses-11-00105-s001.pdf]

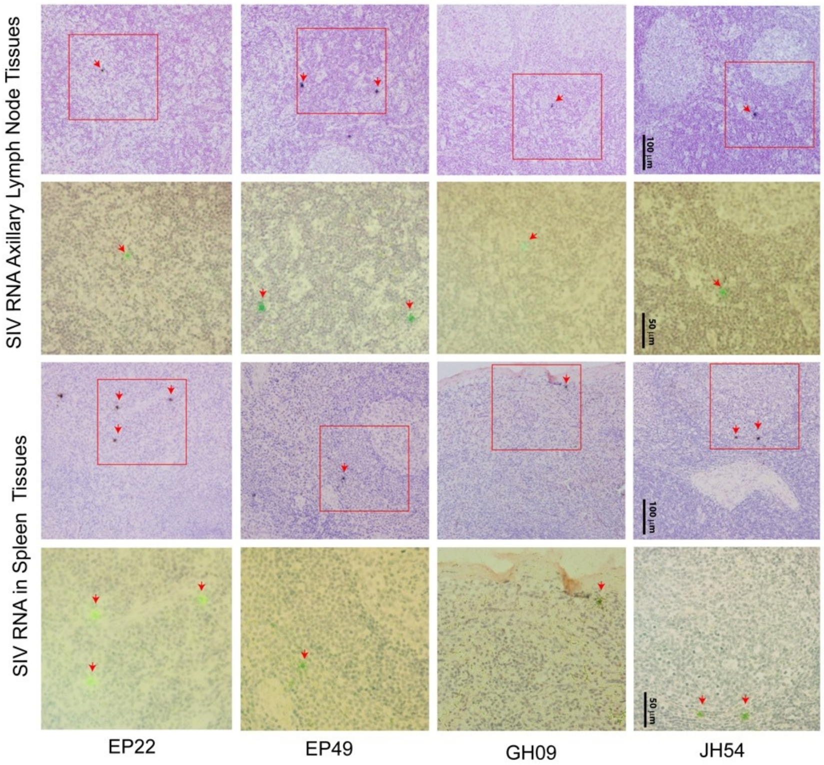

Supplemental Fig 1. SIV viral RNA positive cells in axillary lymph node LN and spleen tissues. Viral RNA+ cells were detected in spleen tissues and axillary lymph node tissues from all 4 aviremic animals using 35S riboprobe in situ hybridization. The frequency of viral RNA positive cells in axillary lymph node tissues are very low, only a few positive cells were detected in whole tissue section. SIV RNA-positive cells were overlaid by silver grains (black in transmitted light shown in the upper panel, and green under epipolarized light shown in lower panel) after radioautography for 14 days. The red box on the upper panel was highlighted in a higher magnificence in the corresponding lower panel.

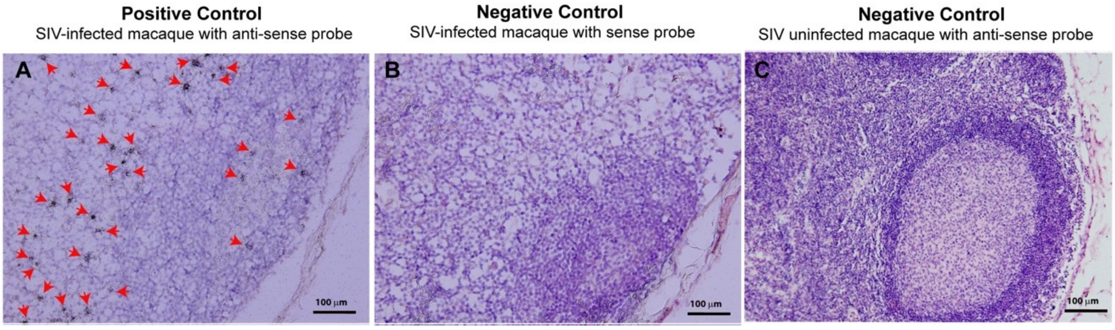

Supplemental Fig 2. Positive and negative control for SIV RNA in-situ hybridization. Isotope 35S labeled anti-sense riboprobe was hybridized to mesenteric lymph node tissues of a rhesus macaque at peak viremia (2-week post infection, animal ID rh4979) as positive control (A), sense riboprobe was hybridized to mesenteric lymph node tissues of above animal as negative control and no signal was detected (B), anti-sense riboprobe was hybridized to mesenteric lymph node tissues of SIV uninfected macaque (animal ID, rh4973) as another negative control and we did not detect any signal (C).
